# Electroconvulsive Therapy in a Renal Transplantation Patient: A Rare Combination of Disease and Treatment

**DOI:** 10.1155/2020/8889883

**Published:** 2020-10-30

**Authors:** Giovanni Malaty, Kerilyn Godbe, Mehdi Elmouchtari, Gurjot Malhi, Justin White, Azziza Bankole, Tracey Criss

**Affiliations:** ^1^Virginia Tech Carilion School of Medicine, 2 Riverside Cr, Roanoke, VA 24016, USA; ^2^Carilion Clinic Psychiatry & Behavioral Medicine, 2017 S Jefferson St, Roanoke, VA 24014, USA

## Abstract

The safety and efficacy of electroconvulsive therapy (ECT) for the treatment of psychiatric disorders have been demonstrated in a wide variety of patients, including postoperative patients and those who are pregnant. While several reports highlight the safety of this treatment in heart and liver transplantation patients, there is a relative lack of literature detailing the safety profile of ECT in an individual with recent kidney transplantation. Here, we explore the case of a patient with a recent renal transplant secondary to diabetes-related end-stage renal disease (ESRD) who underwent a successful course of ECT treatment. A 57-year-old Caucasian male with a past psychiatric history of schizoaffective disorder, bipolar type, and a past medical history of end-stage renal disease with recent right renal transplantation was admitted to the inpatient psychiatry unit. The admission was via a temporary detention order (TDO) for suicidality and auditory hallucinations promoting self-harm. The patient's depressive and delusional history was well-documented and had been refractory to several courses of psychotherapeutic and pharmacologic management. Electroconvulsive therapy was subsequently initiated and was well-tolerated. Treatments progressively alleviated his depressive and psychotic symptoms and did not adversely affect the function of his transplanted kidney, which was closely monitored throughout the treatment process. This case demonstrated the safety and efficacy of ECT treatment in an individual with recent renal transplant and may prompt further trials into establishing safety and efficacy in larger study populations.

## 1. Introduction

Electroconvulsive therapy involves inducing short, generalized seizures using a voltage-generating device to pass an electrode-guided 500-800 milliampere (mA) current through the cerebrum in a bitemporal, bifrontal, or unilateral configuration and is indicated in patients with treatment-resistant depression, mania, psychosis, catatonia, and severe, acute psychiatric changes that occur in pregnancy. Although the precise therapeutic mechanism is not fully understood, the safety profile of ECT has been well-studied in both humans and animals, and the risk involved mirrors that of general anesthesia induction for any other procedure [1]. Other risks are related to seizure activity and include flash pulmonary edema, aspiration pneumonia, dental and tongue injuries, headache, postprocedure disorientation, and memory loss [2, 3]. Efficacy and safety of ECT have been demonstrated in individuals who are pregnant and in those with comorbid conditions who are being treated with glucocorticoids, oral anticoagulation, glycemic control, antacids, antihypertensives, and antiasthma medications [4, 5]. Those with chronic heart conditions such as congestive heart failure, arrhythmias, and hypertension are at risk of symptomatic exacerbation and must achieve preprocedural pharmacologic control of heart rate and blood pressure, as ECT may result in a precipitous rise in both [6].

One specific demographic in which ECT has been well-studied is patients who have undergone organ transplantation prior to initiating treatment, specifically of the heart, and to a lesser degree, the liver. Due to the stressors of chronic disease, transplant patients are at a greater overall risk for developing depression and, in many cases, are treated with psychotropic medication. Reviews of cardiac transplantation patients have almost universally determined that ECT is a safe treatment modality posttransplant and should be considered in this demographic, as those with end-stage heart disease are at an increased risk of depression and psychiatric changes. A 2017 meta-analysis of patients receiving ECT for symptoms of major depression after orthotopic cardiac transplantation showed routine procedural tachycardia and spikes in blood pressure but yielded very few subacute or chronic complications with regard to the health of the transplanted organ [7]. Regarding liver transplantation, only a few cases have been published. Similarly, they yield positive results with regard to symptom remission and report no major complications related to the transplanted organ [8]. A group that is noticeably missing from this list of well-documented demographics includes those who have undergone kidney transplant prior to ECT.

In this case report, we describe a patient with a history of bipolar-type schizoaffective disorder presenting with melancholic depressive symptoms and mood-congruent psychosis who was started on treatment with thrice-weekly bitemporal ECT six months after receiving a right renal allograft for end-stage renal disease secondary to chronic diabetes. He exhibited progressive improvement in mood and decreased recurrence of delusions and hallucinations, with no apparent subacute adverse effects on the function of the transplanted kidney. While several isolated reports exist that detail the interaction between ECT and cardiac and liver transplantation, there is a remarkable paucity of information relating this treatment to recently performed renal transplantation.

## 2. Case Presentation

A 57-year-old Caucasian man with a past medical history significant for ESRD and a psychiatric history notable for bipolar-type schizoaffective disorder presented to the inpatient psychiatry unit via the emergency department. He was admitted on a TDO for two days of worsening depressive symptoms including low mood, hopelessness, feelings of guilt and nihilism, and vague suicidal ideation. Additionally, he exhibited concurrent mood-congruent psychosis with auditory hallucinations encouraging self-harm and delusions of impending personal health catastrophe, personal financial bankruptcy, and outstanding criminal charges. An extensive review of the patient's chart revealed sporadic inpatient psychiatric hospitalizations for depression and benzodiazepine abuse dating back to 2005. Regimens of antidepressant and antipsychotic medication for depression and benzodiazepine abuse were tried since 2011, when he first appeared in our electronic health record after relocating to the area. At that time, he carried the diagnosis of major depressive disorder with appetite changes and low mood and was stable with psychotherapy, fluoxetine, and aripiprazole. Prior medication trials included sertraline, venlafaxine, mirtazapine, hydroxyzine, alprazolam, lorazepam, and clonazepam, without long-term symptomatic relief. In 2017, he was voluntarily admitted to inpatient psychiatry for an acute episode of distractibility, restlessness, pressured speech, sleeplessness, and auditory hallucinations commanding him to commit suicide. His diagnosis was then revised to acute exacerbation of schizoaffective disorder, bipolar type. He was hospitalized for nine days, while being treated for mania and alprazolam dependence, and was discharged home on risperidone, which he has been taking since that hospitalization.

In addition to his well-documented psychiatric history, the patient began experiencing a progressive worsening of diabetes-related chronic kidney disease in 2016, which required the initiation of hemodialysis three times per week. In late 2016, his glomerular filtration rate (GFR) was approximately 14 mL/min (nl ≥ 60 mL/min), and renal ultrasound showed extensive echogenicity, enlargement, and cyst formation in the right kidney. The patient began care with a renal transplant surgeon and was placed on the kidney transplant waiting list, meanwhile continuing pharmacologic treatment and regular dialysis. Three years later, in 2019, a living donor renal allograft of the right kidney was performed without acute complications. Treatment with tacrolimus, mycophenolate, and prednisone was initiated, and the transplant exhibited good function in the subacute phase. His kidney function is monitored regularly, and the patient remains renally stable.

Upon his most recent psychiatric presentation, the patient was overcome with guilt and repeatedly exhibited an overwhelming need to confess to a number of “crimes” he committed over the past six months, which included slandering several acquaintances online, sending his portrait to women he had met on social media, and accessing pornographic content on his computer. He repeatedly admitted that he was fearful but willing to accept any felony charges that would inevitably be brought against him. His spouse regularly communicated with his treatment team and clarified that these events never occurred and that the patient had never been in any legal trouble. The remainder of the patient's delusions was centered around what he perceived as the eventual and inevitable failure of his newly transplanted right kidney, as well as the impending financial difficulty that these health problems would inexorably precipitate. Once again, his spouse assured his treatment team that his transplant surgeon was quite pleased with the kidney's function and that the patient had adequate health insurance to cover any current or eventual medical expenses at the time of presentation.

The patient was being treated with risperidone and venlafaxine at the time of presentation. His delusions and depression persisted despite progressive dose increases of these medications. A judicial authorization was obtained to initiate ECT based on the attending psychiatrist's determination that no other treatment had demonstrated efficacy and that the patient's ongoing suicidality was a major concern. The patient and the family consented to the treatment, and the patient began receiving bifrontal ultra-brief-pulse ECT treatments three times per week. Treatments were conducted under the care of psychiatry and anesthesia in the hospital's designated postanesthesia care unit (PACU) ECT treatment room. Prior to his treatments, a combination of methohexital and succinylcholine was utilized for anesthesia and muscle relaxation, respectively. A Thymatron® System IV Integrated ECT Instrument (Somatics, LLC, Lake Bluff, IL) was used to initiate half-age stimulation at 30% energy. The choice of induction agents, dosages, and ECT stimulation strategy were determined by a consultation between psychiatry and anesthesia per the guidelines of our academic medical center. Following his first treatment, systolic blood pressure episodically rose past 250 mmHg (nl = 120 mmHg), and he was treated with labetalol, which effectively normalized his pressure. During the first two treatment sessions, the patient received postprocedural bilevel positive airway pressure (BiPAP) due to acute respiratory distress. With his fifth treatment, the induction agent was changed from methohexital to etomidate due to a lack of adequate seizure activity. Beginning with his seventh treatment, a clevidipine drip was used for some treatments for the management of occasional significant posttreatment hypertension. Placement of the ECT leads was changed from bifrontal to bitemporal with the patient's eighth treatment due to inadequate clinical seizures at 100% energy. He experienced no headache, memory loss, or other acute neurological complications from these treatments, and his renal function remained stable. The patient was noted to exhibit improvement in his psychosis as early as after three treatments, though his mood did not immediately improve. Following his eighth treatment, his spouse noted that his mood was significantly improved, as the patient was now inquiring about family and neighbors and referring to his delusions much less frequently. He reported little improvement following his ninth session, so the treatment team decided to continue ECT. At the end of treatment twelve, the patient was stable, not exhibiting any paranoia or delusions, and denied suicidal or homicidal thoughts. Prior to discharge, the patient was visibly brighter, more social among his fellow patients, and was actively participating in group therapy sessions, which he refused prior to ECT. Over the subsequent course of his psychiatric hospitalization, the patient reported feeling “happy” and “back on the right track.” His reports of hallucinations and worthlessness noticeably decreased, and he became more open to continued long-term psychiatric counseling. Communication during ECT was maintained with his out-of-state renal transplant surgeon, who was reportedly pleased with the status of his kidney transplant, antirejection medication levels, and blood pressures.

## 3. Discussion

This well-established treatment modality is utilized in the treatment of several psychiatric disorders, including those that feature significant psychomotor impairment and mood-congruent delusions or hallucinations, such as this patient [9]. The exact therapeutic mechanism is still not completely defined; however, current hypotheses center around demonstrated improvement of severe psychiatric symptoms and reversal of deleterious cortical atrophic changes seen in these processes. Efficacy is thought to be derived from some combination of increased monoamine transmission, increased hypothalamic and pituitary hormone activity, and increased structural plasticity and neurogenesis [10, 11]. While no absolute contraindications for ECT exist, patients with chronic cardiac disease are at a higher risk of symptomatic exacerbation. The relation of this treatment to many concurrent pathologies has been well-explored, but a few comorbid conditions have gone relatively underreported. One of these pathologies is end-stage renal disease with kidney transplantation. End-stage renal disease involves a progressive loss of kidney function with end-organ damage, resulting in an increased serum creatinine level and decreased GFR. While a majority of cases are idiopathic, ESRD may also stem from chronic disease processes such as diabetes mellitus, hypertension, nephritic syndromes, or genetic abnormalities such as polycystic kidney disease. A GFR below 15 mL/min is an immediate indication for transplantation, regardless of cause [12].

ECT-induced seizures cause transient blood pressure increases, which are managed periprocedurally by various pharmacologic agents including calcium-channel blockers (CCBs), beta blockers, and nitroprusside. The effects of this management on long-term kidney function, especially in transplanted kidneys, have not been thoroughly explored. In this patient, a long history of type 2 diabetes led to progressive worsening of kidney function, which necessitated hemodialysis. The patient was placed on a kidney transplantation list and received a transplant without subsequent complications. During ECT, regular contact was maintained with the patient's transplant surgeon. During his treatments, creatinine and GFR were monitored and remained stable, and no long-term changes in blood pressure were noted. The patient's transplant medication regimen did not change through his course of ECT, and the patient reported no symptoms of headaches, dizziness, fatigue, or changes in urination, as may be suggested by blood pressure fluctuations and acute renal damage. Periprocedural systolic blood pressures to the 250 s were routinely recorded but were well-controlled with as-needed medication. Both depressive and manic symptoms had been observed along with delusions and hallucinations in this patient. These psychotic symptoms occurred in the absence of depressed mood or mania, and therefore, the diagnosis of schizoaffective disorder was made. Due to the refractory nature of his illness to several types of treatment, ECT was recommended and performed. Twelve treatments of bitemporal ECT progressively improved the patient's mood and abated his psychosis.

Patients with a history of major organ failure with transplantation are at a higher risk for psychiatric illness, specifically depression, and are routinely treated with psychotropic medications [13]. In the case of this patient, his renal dysfunction was preceded by his diagnosis of schizoaffective disorder, meaning that his psychiatric disease was unlikely to be originated by his medical disease. The relationship between ECT and recent kidney transplant is not well-documented in current psychiatric, renal, and surgical literature. While several published reports detail the relationship between ECT and heart or liver transplantation, there is a relative lack of published information on this specific reported disease-therapy combination [7, 8]. Following a thorough review of the current literature, only one case has been identified; however, it does not extensively detail the resultant effects, if any, on the transplanted kidney [14]. The full safety profile for ECT in patients who have undergone kidney transplantation is yet to be elucidated. While ECT has been declared relatively safe in patients with heart and liver transplants, relatively little information exists detailing the safety of this treatment in renal transplant. This case has demonstrated that ECT was both efficacious and safe in at least one patient with the unique combination of pathologies. Hopefully, these findings influence providers to use ECT with confidence in individuals with these medical histories, so that more data regarding this therapy can be collected, and more patients can benefit from this highly efficacious treatment.
